# Non-Traditional Risk Factors as Contributors to Cardiovascular Disease

**DOI:** 10.31083/j.rcm2405134

**Published:** 2023-04-28

**Authors:** Lina Wang, Jingshu Lei, Ruiying Wang, Kuibao Li

**Affiliations:** ^1^Department of Cardiology, Hebei Yanda Hospital, 065201 Langfang, Hebei, China; ^2^Department of Hematology, Hebei Yanda Lu Daopei Hospital, 065201 Langfang, Hebei, China; ^3^Heart Center & Beijing Key Laboratory of Hypertension, Beijing Chaoyang Hospital, Capital Medical University, 100016 Beijing, China

**Keywords:** cardiovascular disease, cardiovascular risk, gut microbiota, sleep disorder, psychosocial factors, Vitamin D deficiency, environmental exposure, hyperhomocysteinemia, hyperuricemia

## Abstract

Cardiovascular disease (CVD) remains one of the primary causes of morbidity and mortality worldwide. Classic cardiovascular risk factors, such as hypertension, diabetes mellitus (DM), hyperlipidemia, and smoking, have been well identified and given increased attention in clinical practice. However, the incidence and prevalence of CVD remains high, especially in developing countries. Therefore, there has been more attention to non-traditional CVD risk factors such as gut microbiota, sleep disorders, dietary structure, and psychosocial factors in their important roles in the development of CVD. In this review we summarize the association of non-traditional risk factors with CVD with the aim of further reducing the risk of CVD.

## 1. Introduction 

**Fig. 1.**
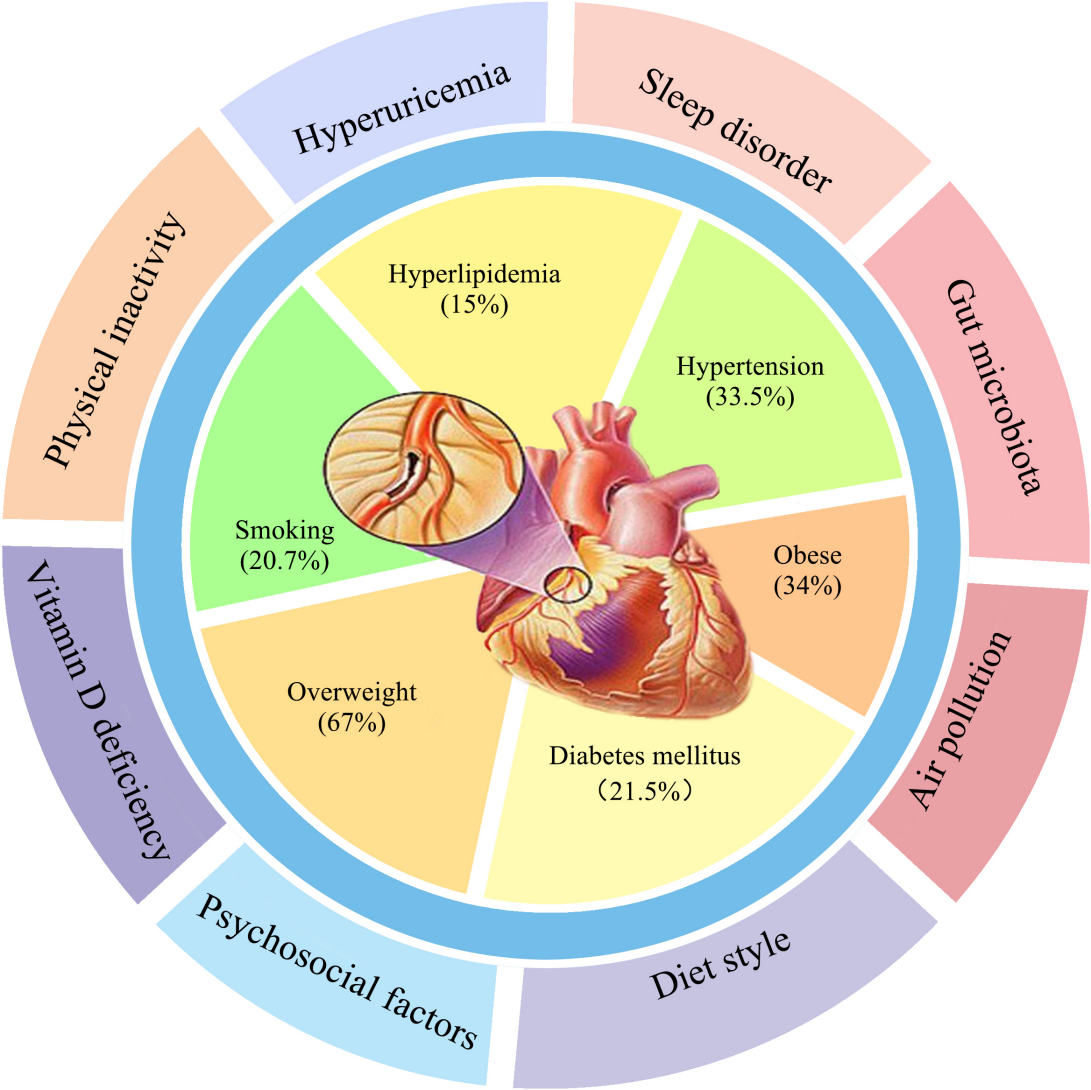
**The first circle is traditional CVD factors, and the second circle is new non-traditional CVD risk factors**.

Cardiovascular disease (CVD) remains a major public health issue and one of the primary causes of morbidity and mortality worldwide [1]. The prevalence of well-known modifiable cardiovascular risk factors include hypertension (33.5%), smoking (20.7%), hyperlipidemia (15%), diabetes mellitus (21.5%), overweight (67%) and obesity (34%) [2, 3] (Fig. 1). Age and male sex are traditional non-modifiable risk factors. The multicenter case-control INTERHEART study showed that common risk factors accounted for 90% of the population attributable risks (PAR) in men and 94% in women with myocardial infarction, and included smoking (PAR 35.7%), increased apolipoprotein B/apolipoprotein A1 (ApoB/ApoA1) ratio (PAR 49.2%), hypertension (PAR 17.9%), diabetes (PAR 9.9%), and abdominal obesity (PAR 20.1%) [4]. However, these traditional risk factors by themselves cannot explain the increased incidence of CVD. It is known that 25% of patients with premature CVD and 12% of patients with an acute myocardial infarction, do not have any established risk factors [5, 6].

Despite great efforts to control these conventional risk factors, there are still residual cardiovascular risks. These may be largely due to “non-traditional” cardiovascular disease factors, which have been identified based on studies of the pathogenesis of atherosclerosis and atherothrombotic cardiovascular events since 1999 [7]. These risk factors include chronic inflammation and its markers, such as C-reactive protein, oxidative stress and endothelial dysfunction; lipoprotein (a) [Lp(a)]; psychosocial factors, such as environmental stress and responsiveness to stress; plasma insulin levels and markers of insulin resistance; and activation of the renin-angiotensin system [7]. However, the strength of the associations of these risk factors with CVD events and therapy still need to be defined [7]. The 2018 US Preventive Services Task Force studies of asymptomatic adults with no known cardiovascular disease, found that non-traditional CVD risk factors such as ankle brachial index (ABI), high-sensitivity C-reactive protein (hsCRP) levels, and coronary artery calcium (CAC) scores, were not independent factors for CVD [8]. Recently, Whayne *et al*. [9] summarized the non-Traditional CVD risk markers as ApoA, ApoB, hsCRP, homocysteine, interleukin 1 (IL1), Lp(a), the density of low-density lipoprotein (LDL) particles, the LDL particle number, tissue/tumor necrosis factor-α (TNF-α) and uric acid. It has been thought that non-traditional risk factors such as gut microbiota, sleep disorder, dietary structure, air pollution and psychosocial factors may contribute to increased CVD risk. These novel risk factors may have an important role in the development of CVD. The importance of non-traditional risk factors for the prevention of CVD is gaining increasing attention. In this article we review these major non-traditional CVD risk factors.

The traditional risk factors refer to the risk factors that are well proven and have a clear relationship with CVD, such as hypertension, diabetes, dyslipidemia, obesity, and smoking. The non-traditional risks discussed in this article include relatively common “new” risk factors, such as gut microbiota, vitamin D deficiency, and lack of exercise (Fig. 1). Non-traditional risk factors with unclear relationships, such as COVID-19, and rare non-traditional risk factors such as poly genomic scores, pregnancy related complications, IL1, and TNF-α will not be reviewed.

## 2. Non-Traditional Factors Indirectly Contribute to the Increased Risk of CVD

### 2.1 Gut Microbiota

**Fig. 2.**
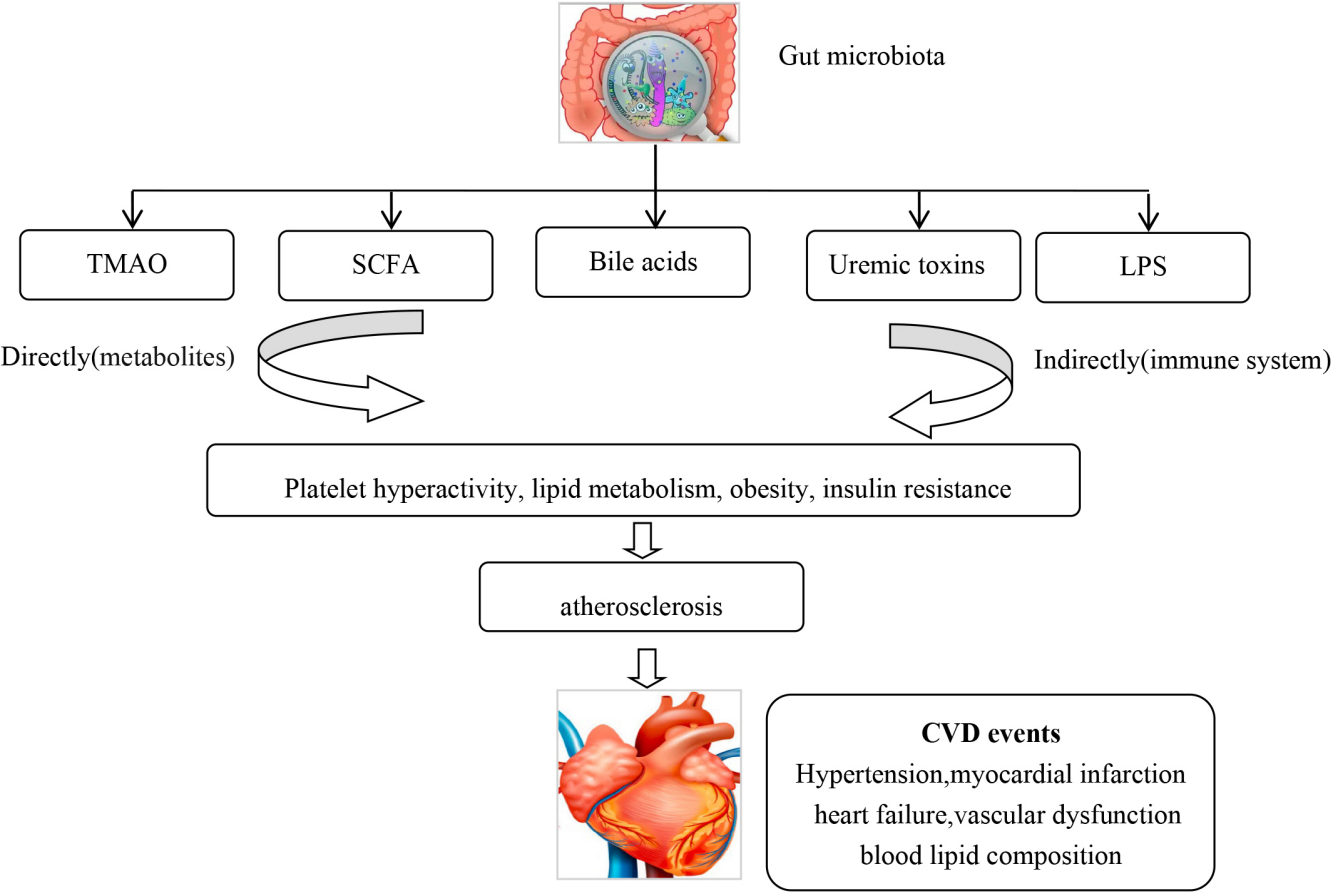
**The gut microbiota can via metabolites and the immune system lead to CVD**. CVD, Cardiovascular disease.

The human gut is inhabited with approximately 100 trillion bacteria, which can modulate both physiology and body metabolism [10]. Emerging evidence has showed an association of gut microbiota and their metabolites with CVD. Asymptomatic and symptomatic atherosclerotic plaques contain different pathogenic microbiomes [11], such as Pseudomonas, Streptococcus and Chlamydia pneumoniae [12, 13, 14]. Studies have found that specific gut microbial species are associated with inflammation and atherosclerosis [15], hypertension and vascular dysfunction [16, 17], symptomatic stroke and transient ischemic attack [18], heart failure [19] and blood lipid composition [20]. Pathogenic bacteria can make vessel walls more vulnerable to atherosclerotic plaque formation, either by infection or indirectly by an auto-immune inflammatory reaction [21, 22]. Gut microbiome was associated with an increased incidence of acute myocardial infarction (AMI) in animal models [23]. Gut microbiota can generate trimethylamine N-oxide (TMAO) [24], short chain fatty acids (SCFA) [25], uremic toxins [26], bile acids [27], and lipopolysaccharides (LPS) [28], which can alter the hosts metabolism. TMAO increases CVD risk by affecting platelet hyperactivity, lipid metabolism, obesity, and insulin resistance [29, 30]. TMAO and phenylacetylglutamine (PAGln) have been shown to induce platelet hyper-reactivity and increase thrombotic formation [29]. TMAO has been associated with an increased incidence of CVD and could predict the risk for myocardial infarction (MI), stroke or death even after adjustment for traditional CVD risk factors [30, 31]. TMAO has also been associated with an increased risk of atrial fibrillation [32]. SCFAs regulate blood pressure by the angiotensin-renin system pathway [33, 34]. Lipopolysaccharide-binding protein (LBP) levels have been found to be significantly higher in coronary artery disease (CAD) patients and was shown to be an independently biomarker for total and CVD related mortality [35]. A cohort study on stools from 218 patients with atherosclerotic cardiovascular disease (ACSVD) and 187 healthy controls demonstrated that gut microbiome in ASCVD patients had an increased abundance of Enterobacteriaceae and *Streptococcus *spp. [36]. CVD and diabetic medication may also effect gut microbiota, which may alter the absorption of these drugs, especially lipid lowering and diabetic medications [37, 38, 39] (Fig. 2).

Age, sex, ethnicity, diet, and lipid levels may also affect gut microbiota and add to the difficulty in determining the role of gut microbiota in CVD [40]. Although accumulated evidence shows gut microbiota and their metabolites play a vital a role in inflammation and CVD [41], a recent meta-analysis found that antibiotic treatment had no beneficial effect on the risk of CVD [42]. Further work is required to establish the role of gut microbiota in preventative and therapeutic regimes to reduce the risk of CVD.

### 2.2 Physical Inactivity

Physical inactivity is an import risk factor for numerous metabolic diseases. About 1.9 million deaths annually are estimated to be due to physical inactivity [43] and $117 billion healthcare dollars annually are spent on diseases attributed to inadequate physical activity [44]. The risk of CVD increases in individuals with physical inactivity and a sedentary lifestyle [45]. It has been estimated that physical inactivity is responsible for 6% of CVD [46]. There is evidence for a relationship between increased physical activity and a lower incidence of CVD and mortality, conversely greater amounts of sedentary behavior are linked to an increased risk of CVD and mortality [44]. Sedentary behavior in CVD patients increases the risk of depression and leads to insufficient physical activity [47]. A meta-analysis showed that reducing sedentary behaviors result in an increase in life expectancy [48]. A study examining sitting time and mortality in 17,013 Canadians showed a dose-response relationship between sitting time and all-cause mortality and CVD. There was a 54% increased risk of CVD mortality among adults with sitting “almost all of the time” compared to those with sitting “almost none of the time” [49]. Conversely, a meta-analysis including 160 randomized controlled trials with 7487 participants revealed that exercise could lower fasting insulin, triglycerides, Glycosylated Hemoglobin-type A1C (HbA1c), leptin, fibrinogen, and angiotensin II, and raised high-density lipoprotein cholesterol, apolipoprotein A1, and interleukin-18 [50]. In addition, 17 meta-analyses and one systematic review with 594,129 adults confirmed that physical activity reduces blood pressure on a dose-response relationship among adults with normal range blood pressure, prehypertension, and hypertension [51].

The strong evidence from these studies supports the premise that adults should reduce their sedentary time to less than 9 hours per day, or even less than 6–8 hours per day [52, 53]. Physical activity is one of the most important modifiable risk factors, which can decelerate the atherosclerotic process, control CVD risk factors, such as hypertension, diabetes, and obesity [54]. More than 7000 steps/day or even 10,000 steps/day are recommended for the secondary prevention of CVD to achieve a decrease in blood pressure and BMI [55, 56, 57]. A meta-analysis which included six studies with 693 patients suggested self-monitoring of physical activity by patients with CVD had a significant effect on decreasing the risk of CVD [58]. The 2018 Physical Activity guidelines recommended at least 150–300 minutes per week of moderate intensity aerobic exercise, or 75–150 minutes of vigorous exercise for adults and those exceeded this level by at least 3–5 times continued to show a 40% reduction in CVD mortality [44].

### 2.3 Diet Style

There is a strong correlation between diet and incidence of CVD. Diets rich in saturated fat and cholesterol are the major cause of CVD and atherosclerosis, and have been termed “the diet–heart hypothesis” [59]. This has led to numerous diets to alter the risk of CVD. The Mediterranean diet has been found to lower blood pressure, fasting glucose, and blood lipids [60]. The Lyon Diet Heart Study, enrolling 505 patients with a myocardial infarction, showed that patients assigned to the Mediterranean diet had a lower incidence of cardiac deaths, overall mortality, and nonfatal myocardial infarctions [61]. A study enrolling patients with acute coronary syndromes showed that a Mediterranean diet was associated with preserved left ventricular systolic function, more favorable myocardium remodeling, and a decreased incidence of recurrent CVD events [62]. A beneficial effect of a Mediterranean diet on diastolic and systolic function was also demonstrated in patients with chronic heart failure (CHF) [63]. A large prospective, multicenter cohort study of Spanish university graduates with an average follow-up of 11.5 years, demonstrated that compliance with the Mediterranean diet and lifestyle significantly lowered the risk of CVD [64]. Alternative diet styles have also been studied. A 10 year cohort study of Greek adults found no correlation between a dietary approaches to stop hypertension (DASH)-style diet and the risk of CVD on long-term follow-up [65]. In contrast, a meta-analysis of prospective cohort trials found that compliance with the DASH diet showed a 20% decrease in the incidence and mortality in CVD [66].

A ‘High-salt’ diet has been shown to increase the risk of CVD [67]. A cross-sectional study including 2632 coal miners showed that ‘High-salt’ and ‘Refined grains’ diets significantly increased 10-year atherosclerotic CVD risk scores and 10-year ischemic CVD risk scores [68]. Refined foods can be digested rapidly with none of the fiber-rich structure of high-starch vegetables. However, it has been reported that a refined food diet is associated with an increased risk of CVD [69].

Dietary habits are an important adjustable factor in the prevention of CVD [70]. Compliance with the Mediterranean dietary pattern can significantly decrease the incidence of CVD and its adverse events [64].

## 3. Non-Traditional Factors Associated with Increased Risk of CVD 

### 3.1 Sleep Disorders

Sleep disorders are an important health issue. Approximately 30% of the general population has a sleep related condition [71]. A series of meta-analyses have confirmed both too little (<7 hours) and too much sleep (>9 hours) could increase the risk of CVD [72, 73, 74]. The cross-sectional study of the NHANES survey enrolled 32,152 participants in a sleep study from 2005 to 2016. The results of this study showed that shorter sleep duration (<7 hours) was related to a higher incidence of DM, hyperlipidemia, and stroke, while longer sleep (>9 hours per night) duration was associated with a higher incidence of CAD, stroke, and heart failure [75]. The quality of sleep and insomnia can also affect cardiovascular risk. The Sleep Heart Health Study, including 4994 participants with a median of 11.4 years follow-up, showed that participants with insomnia or poor sleep habits had a 29% higher risk of CVD in contrast to participants without insomnia or poor sleep habits and an objective sleep time of at least 6 hours [76]. A prospective cohort study including 60,586 adults aged ≥40 years demonstrated that participants <6 h/d sleep duration had a significantly increased risk of coronary heart disease (CHD) (HR: 1.13, 95% CI: 1.04–1.23). Poor sleep quality, dreamy sleep (HR: 1.21, 95% CI: 1.10–1.32) and difficulty falling asleep (HR: 1.40, 95% CI: 1.25–1.56) were also associated with an increased risk of CHD [77]. Poor sleep quality is highly prevalent in patients with CVD [78]. Patients with poor sleep quality have a higher risk for CVD, including acute myocardial infarction and heart failure [79, 80, 81]. Insomnia results in a higher nighttime systolic blood pressure (SBP) as well as slower drop in nocturnal blood pressure, which increases the incidence of CVD [82]. Insomnia was also found to be associated with increased cardiovascular mortality [83]. A meta-analysis of 74 studies with 3,340,684 participants demonstrated that those who failed to follow the recommended 7 to 8 hours of sleep had a higher risk of CVD events and mortality [84].

The mechanisms responsible for the association of sleep disorders with CVD may be attributed to increased appetite and food intake, and altered glucose intolerance, and metabolic changes [85]. In addition, longer sleep duration is related to poor lifestyle behaviors, such as unhealthy diets, decreased exercise, increased psychologic disorders, and impaired metabolic syndromes, all of which increase the incidence of heart failure and stroke [86]. Sleep disorders have emerged as a target for interventions to reduce the incidence and risk of CVD.

### 3.2 Psychosocial Factors

Psychosocial stress includes stress symptoms and stressors, such as loneliness and critical life events. The WHO estimated the prevalence of depression among CVD patients at 3%–9% worldwide [87]. It was especially higher in China and Iran, with a prevalence of 35% to 47% [88, 89]. Seldenrijk *et al*. [90] found that depression had a 3-times higher risk for progression to CVD during a six-year follow-up. The INTERHEART study showed self-reported psychosocial factors were independently associated with the risk for an acute MI [91]. Depression has been found to be another non-traditional risk factor for cardiovascular morbidity and mortality in patients with CHD [92]. Depression can result in deterioration of heart function [93]. A meta-analysis including 10,785 AMI patients demonstrated that approximately 20% suffered from severe depression, and one of three patients had mild to moderate depression [94]. Another meta-analysis of 30 prospective cohort trials found that depression was independently associated with an increased risk of CAD and AMI [95]. On the other hand, CVD is also associated with an increased risk of depression [96]. Up to 40% of patients who had a major cardiovascular event also had major depression [97]. Fotopoulos *et al*. [98] found that over half of patients with myocardial disease (53.8%) had depression, anxiety, or both.

Studies have also demonstrated a dose-dependent relationship between the severity of depression and anxiety and CVD [99]. Both anxiety and depression have been shown to be an associated with AMI [100, 101]. Acikel *et al*. [102] reported that excellent management of psychological status during and post cardiac surgery could improve quality of life and cardiovascular prognosis. Depression and anxiety are also often observed in CHF patients [103]. Approximately 60% of heart failure (HF) patients have anxiety and 30% suffer from depression [104, 105, 106]. Depression or anxiety were associated with a lower exercise capacity in HF patients [107, 108]. Anxiety was also associated with rehospitalization in CHF patients [109]. Stress can also influence cardiovascular health. Studies have shown that men are more likely to be faced with job-related stress; while women more frequently face chronic stress, both of which are related to CVD risk [110, 111, 112]. In addition, women had higher anxiety levels compared with men [113]. Serpytis *et al*. [114] demonstrated that women were at an increased risk of anxiety and/or depression in comparison to men in patients with a myocardial infarction. Several studies have demonstrated that women were more likely to suffer from psychological stress during and after the recent pandemic. This may be attribute to preexisting depressive and anxiety disorders, chronic environmental strain, and domestic violence, which leads to sedentary behavior and deteriorating lifestyle habits. In addition social, economic, and cultural factors may also contribute to the effect of gender on CVD [115, 116, 117].

The exactly mechanism of mental disorders increasing the risk for CVD remains uncertain. Currently it is known that psychosocial stress has direct biological effects and is indirectly correlated with socioeconomic and behavioral risk factors [118]. This directly effects the amygdala-based fear-defense system and other pathophysiological pathways [119]. Psychosocial risk factors can also activate the sympathetic system and hypothalamic-pituitary-adrenal axis resulting in endothelial dysfunction, and generation of proinflammatory cytokines, platelet activation, and activation of cardiogenic sympathetic nerves, which leads to an increased heart rate, myocardial infarction, arrhythmias, and even sudden death [113].

### 3.3 Vitamin D Deficiency

Almost 23% of the Australia population, 24% of USA, 37% of Canada, 6%–76% of Europe, and 6–70% of South East Asia suffer from vitamin D deficiency (25(OH)D level <50 nmol/L) [120, 121]. In patients with coronary artery disease, the incidence of vitamin D deficiency ranged from 80 to 95% [122, 123].

Vitamin D deficiency is another CVD risk factor, which could cause ventricular hypertrophy, arterial stenosis, heart failure and arrhythmias [124]. The relationship between vitamin D levels and MI, HF, coronary artery disease (CAD) and stroke is significant (*p *< 0.0001) [125]. A randomized prospective, case-control study measured 25(OH)D levels in 294 patients diagnosed with CHD and 438 matched controls. The mean level of 25(OH)D was 13.12 ± 11.13 and 18.28 ± 8.34 respectively (*p* = 0.036). This study demonstrated that the lower levels of vitamin D is associated with a higher risk of CHD [126]. The Multi-Ethnic Study of Atherosclerosis (MESA) [127] included 6436 participants measuring baseline serum 25(OH)D concentrations, after a median follow-up of 8.5 years. CHD events occurred in 361 participants; a lower 25(OH)D concentration was associated with a greater risk of CHD among participants who were white (HR: 1.26, 95% CI: 1.06–1.49) or Chinese (HR: 1.67, 95% CI: 1.07–2.61). Similarly, Zhernakova *et al*. [124] demonstrated that vitamin D deficiency was an independent risk factor in elderly myocardial infarction patients. Nepal *et al*. [128] also revealed that 64.7% of patients with acute coronary syndrome suffered from vitamin D deficiency. A non-linear Mendelian randomization analysis showed CVD risk initially decreased dramatically with a rise of 25(OH)D concentrations and plateaued at around 50 nmol/L [129]. Recently, a stratified Mendelian randomization genetic analysis including 386,406 participants suggested there was a significant dose-response relationship between 25(OH)D concentrations and CHD, stroke, and mortality [130]. A meta-analysis that included 283,537 participants, from eight unique prospective cohorts including individuals with 25(OH)D levels measured at baseline and studies where vitamin D status was evaluated according to dietary intake, showed that participants with the highest 25(OH)D levels had a 30% lower risk of hypertension [131].

However, not all the data is consistent with a beneficial effect of Vitamin D on the reduction of CVD. Several RCTs and meta-analyses failed to confirm the cardiovascular benefit with vitamin D supplements. Mendelian randomization studies did not imply a causality between genetically predicted 25(OH)D concentrations and CVD outcomes [132]. A systematic review conducted in 2010 did not confirm the association between vitamin D and cardiometabolic outcomes [133]. In addition, several well-designed trials also did not confirm cardiovascular benefits from vitamin D supplements. One randomized controlled trial (RCT) of 5108 community dwelling residents aged 50 to 84 who were randomized to vitamin D3 versus placebo, found that during a median of 3.3 years, 25(OH)D concentrations increased by more than 20 ng/mL compared with placebo, but this did not result in a reduction in the primary endpoint of the incidence of CVD and death (HR: 1.02, 95% CI: 0.87–1.20) [134]. Another large randomized double-blind, placebo-controlled study, Vitamin D Assessment (VIDA), found that vitamin D supplements did not have beneficial effects on the cumulative incidence of CVD [135]. Meta analyses of RCTs also have not showed a reduction in BP with vitamin D supplements [136]. This trial enrolled 25,871 participants without CVD at baseline, randomized to a 2000 IU/daily dose of vitamin D3 versus placebo. During a mean follow-up period of 5.3 years, there was no significant reduction in CVD events (HR: 0.97, 95% CI: 0.85–1.12) for vitamin D compared with placebo [137].

Vitamin D signaling is thought to alter the pathophysiology of atherosclerosis by reducing the expression of TNF-α, IL-6, IL-1, and IL-8 in isolated blood monocytes that modulate the inflammatory response and by increasing nuclear factor-κB (NF-κB) [138, 139]. The detrimental effects of low levels of vitamin D on CHD, may be attributed to increased inflammation and increased platelet activation [140]. In summary, there is inconsistent evidence from experimental and clinical trials on the role of vitamin D in the pathophysiology of cardiovascular health (Table 1, Ref. [124, 125, 126, 127, 128, 129, 130, 131, 132, 133, 134, 135, 136, 137]). Because of these limitations, vitamin D cannot be recommended for the prevention of CVD events [141].

**Table 1.** **Summary of major studies evaluating Vitamin D deficiency and CVD events**.

Author	Publication year	Study type	No. Patients or studies	Central message
N I Zhernakova [124]	2021	Case control study	187	The deficit level of D-(25(OH)D) should be considered a laboratory predictor of MI in the elderly.
Jeffrey L Anderson [125]	2010	Observational study	41,504	Vitamin D might play a primary role in CV risk factors and disease.
Hamidreza Norouzi [126]	2019	Randomized prospective, case-control study	732	Lower levels of vitamin D is associated with increased risk and extent of coronary artery involvement as well as some of the risk factors of CAD, including male gender, hypertension and positive family history for CVD.
Cassianne Robinson-Cohen [127]	2013	Prospective cohort study	6436	Lower serum 25(OH)D concentration was associated with an increased risk of incident CVD events among participants who were white or Chinese but not black or Hispanic.
Richa Nepal [128]	2021	Descriptive cross-sectional study	51	Prevalence of vitamin D deficiency among patients of ACS was comparable to various other homologous international studies.
Ang Zhou [129]	2022	Non-linear Mendelian randomization	295,788	Vitamin D deficiency can increase the risk of CVD. Burden of CVD could be reduced by population-wide correction of low vitamin D status.
Emerging Risk Factors Collaboration/EPIC-CVD/Vitamin D Studies Collaboration [130]	2021	Observational analyses	386,406	Stratified Mendelian randomisation analyses suggest a causal relationship between 25(OH)D concentrations and mortality for individuals with low vitamin D status.
Setor Kwadzo Kunutsor [131]	2013	Meta-analysis	283,537	Participants with the highest 25(OH)D levels had a 30% lower risk of hypertension.
Tao Huang [132]	2019	Mendelian randomisation	205,923	No evidence to support that genetically increased 25(OH)D was associated with a lower risk of ischaemic stroke, intracerebral haemorrhage, subarachnoid haemorrhage, and lipid levels in both Chinese and European adults.
Anastassios G Pittas [133]	2010	Systematic review	13 observational studies (14 cohorts) and 18 trials	The association between vitamin D status and cardiometabolic outcomes is uncertain. Trials showed no clinically significant effect of vitamin D supplementation at the dosages given.
Robert Scragg [134]	2017	A Randomized Clinical Trial	5108	Monthly high-dose vitamin D supplementation does not prevent CVD.
RKR Scragg [135]	2019	A randomised, double-blind, placebo-controlled trial	5110	Vitamin D supplements did not have beneficial effects on the cumulative incidence of CVD.
JoAnn E Manson [137]	2019	A nationwide, randomized, placebo-controlled trial	25,871	Supplementation with vitamin D did not result in a lower incidence of invasive cancer or CVD events than placebo.
Dongdong Zhang [136]	2020	Meta-analysis	11 cohort studies and 27 RCTs, with 43,320 and 3810 participants	Supplementation with vitamin D does not lower blood pressure in the general population.

CVD, Cardiovascular diseases; ACS, acute coronary syndrome.

### 3.4 Hyperuricemia

Hyperuricemia is defined as serum urate concentrations >7 mg/dL (>420 μM) in men and >6 mg/dL (>360 μM) in women [142]. The prevalence of hyperuricemia has remained at a relatively high level, 20.2% in men and 20.0% in women [143]. The causal relationship between hyperuricemia and CHD has been discussed in many epidemiological studies.

Prospective cohort studies indicated that the levels of uric acid were independently and significantly related to cardiovascular mortality [144, 145]. High levels of uric acid (UA) are also related to an increased incidence of hypertension. A meta-analysis including 97,824 participants showed that hyperuricemia could increase the risk of hypertension [146]. In addition, a cross-sectional cohort of 285,882 individuals demonstrated that UA levels were significantly associated with atrial fibrillation (AF) [147]. A meta-analysis confirmed that high UA was associated with AF in both cross-sectional and cohort studies [148]. Hyperuricemia is also seen in patients with HF. A Japanese registry study showed that hyperuricemia frequently occurred in HF patients and that higher UA levels were associated with increased adverse long-term outcomes [149], suggesting the level of UA is a prognosis related marker in patients with HF.

Hyperuricemia also plays a significant role in other cardiovascular diseases. Abbott *et al*. [150] studied 5209 subjects who developed CHD in the Framingham Study and found that the two-year incidence of gout was 6 times greater in men in comparison to women during 32 years of follow-up. However, some analyses have reported contrary conclusions [151, 152]. In a study including 63,127 participants without a history of CVD, after a median follow-up of 7.04 years, there was no significant relationship between changes in SUA and CVD [153]. In contrast, a meta-analysis involving 341,389 adults showed that for every 1 mg/dL increase in UA, the incidence of CHD and all-cause mortality increased by 20% and 9%, respectively [154]. Another meta-analysis involving almost one million patients (n = 949,773) diagnosed with gout demonstrated the prevalence of MI was 2.8%, HF was 8.7% and hypertension was 63.9%; and that the risk of CVD was higher in subjects with gout compared to non-gout controls [155]. Of note, some studies have suggested a U-shaped relationship between UA levels and CVD [156]. Li *et al*. [157] in a cohort study of 82,219 participants demonstrated that the age at hyperuricemia onset was an important predictor of CVD and all-cause risk for mortality.

Studies have shown that hyperuricemia could induce inflammation, oxidative stress, and endothelial dysfunction, which directly or indirectly increases the risk for CVD [158].

### 3.5 Environmental Exposure

Air pollution is a global health care challenge that has harmful effects on the cardiovascular system. Almost 20% of CVD deaths globally were attributed to air pollution [159]. Either short-term or long-term exposure to air pollutants can result in cardiac dysrhythmias, due to oxidative stress, autonomic dysfunction, coagulation dysfunction and inflammation [160].

Among diverse air pollutants, particulate matter (PM) plays a major role in CVD, especially when its diameter is equal or less than 2.5 μm (PM2.5). Pan *et al*. [161] in Kaohsiung city, found PM2.5 was related to a rise of 25% (95% CI: 2.6%–53.4%) in the risk of STEMI emergency department visits. The Pan-Asian Resuscitation Outcomes Study (PAROS) study found moderate and unhealthy levels of Pollutant Standards Index (PSI) were significantly associated with an increased incidence of cardiac arrest [162]. HF hospitalizations and death have been definitely established in a meta-analysis of 35 studies demonstrating a 2.12% increase after short-term exposure to 10 μg/$m^{3}$ of PM2.5 (95% CI: 1.42–2.82) [163]. A case-crossover study involving 2,202,244 hospitalized patients with CVD demonstrated critically heavy PM2.5 pollution events continuing for three days or more resulted in a substantially increased hospital admission risk for total CVD, angina, MI, ischemic stroke, and HF [164]. Similarly, a large scale multicenter study including 8,834,533 hospitalized patients with CVD in 184 Chinese cities showed that PM2.5 was related to increased hospitalization for ischemic heart disease, HF, arrhythmia, and ischemic stroke [165]. A prospective 10-year cohort study confirmed PM air pollution was associated with progression of coronary calcification and acceleration of atherosclerosis [166]. Short-term PM2.5 exposure may also contribute to acute coronary events [167]. A meta-analysis including 34 studies revealed that all the main air pollutants were significantly associated with an increased for an MI [168]. The reduction in STEMI cases during the COVID-19 pandemic lockdown, was associated with a reduction in polluting emissions [169].

The possible mechanism for the harmful effects of environmental pollution results from the fact that even a short duration of exposure to PM2.5 could result in initiation of oxidative stress or inflammation, autonomic imbalance, and translocation of PM mixture components into the systemic circulation [170], which in turn increases heart rate, blood pressure, plaque vulnerability, and thrombosis formation [164], leading to plaque formation and ultimately rupture and acute coronary syndromes [171].

## 4. Non-Traditional Factors Related to the Current CVD Intervention and Prevention

Non-traditional factors indirectly contribute to CVD. Control of these factors will reduce the numbers of traditional CVD risk factors and contribute to a lower prevalence of diabetes, lower blood pressure, and lower cholesterol levels, which would ultimately lead to better prevention of heart attacks, strokes, and mortality from CVD. This supports the current prevention and intervention guidelines that recommend lifestyle modification, diet and increased physical activities, in an effort to reduce the incidence and risk from CVD. Non-traditional factors have been associated with an increased risk of CVD (Table 2), such as sleep disorders and environmental and psychosocial factors such as depression. Therefore, reducing air pollution exposure, controlling negative psychosocial factors, ensuring proper sleep duration and optimal sleep quality, play an importance role in reducing the risk of CVD. Non-traditional factors can be a possible explanation for the residual risk of CVD in patients with coronary heart disease who do not have any traditional risk factors.

**Table 2.** **Current evidence between non-traditional factors and CVD**.

Risk factors	Consistency of conclusions	Effectiveness after Intervention
Gut microbiota	Not confirm	Not sure
Physical inactivity	Consistency support	Yes
Diet style	support	Yes
Sleep disorders	support	Yes
Psychosocial factors	support	Yes
Vitamin D deficiency	Not confirm	Not confirm
Hyperuricemia	support	Yes
Environmental exposure	support	Yes

CVD, Cardiovascular diseases.

Therefore, it may be appropriate to update current guidelines to include non-traditional risk factors as significant risk factors for CVD.

## 5. Conclusions

Several studies have established the relationship between non-traditional risk factors and CVD. These non-traditional risk factors include intestinal flora dysbiosis, sleep disorders, psychosocial stress, physical inactivity, vitamin D deficiency, hyperuricemia, air pollution, and poor diets. Furthermore, ethnicity [172], socioeconomics [173], chronic pain [174], obesity [175], and air pollution [176] have also been reported to be associated with CVD. Since most non-traditional risk factors are identified as important modifiable factors in CVD prevention, we should recognize all of these reversible risk factors for each patient and provide them with personalized preventive and therapeutic strategies to further reduce the risk for CVD and its adverse events.
